# Onset of polyarticular juvenile idiopathic arthritis with both anti-cyclic citrullinated peptide antibodies and rheumatoid factor in a 3-year-old girl

**DOI:** 10.1186/1546-0096-10-41

**Published:** 2012-12-13

**Authors:** Kozo Yasui, Sonoko Sakata, Hideaki Ochi, Shinji Itamura, Kenta Hirai, Mieko Takenaka, Osamu Mitani, Kazunori Ogawa, Kuniaki Iyoda

**Affiliations:** 1 Department of Pediatrics, Hiroshima City Hospital, 7-33 Moto-machi, Naka-Ku, Hiroshima, 730-8518, Japan

**Keywords:** Anti-CCP antibody, Rheumatoid factor, JIA, Tocilizumab

## Abstract

This report describes 3 year old girl with the unusual presentation of polyarticular juvenile idiopathic arthritis (JIA) with anti-cyclic citrullinated peptide (anti-CCP) antibodies and a positive rheumatoid factor (RF). She was initially treated with a nonsteroidal anti-inflammatory drug (NSAID; ibuprofen) followed by methotrexate (MTX, 10 mg/m^2^/week) and prednisolone (0.25 mg/kg/day), but these treatments were ineffective. Administration of tocilizumab, a humanized antihuman interleukin-6 receptor monoclonal antibody, promptly improved her clinical manifestations, and she has been in complete remission (DAS28 <2.6) without bone erosion and/or destruction. Positivity for both antibodies (anti-CCP and RF) can forecast the severity of JIA (radiographic bone destruction). In such cases the administration of biologic remissive therapy may be prudent early in the disease course.

## Background

Juvenile idiopathic arthritis (JIA), previously called “juvenile chronic arthritis” or “juvenile rheumatoid arthritis”, is the most common chronic autoimmune (auto-inflammatory) disease in childhood. The disease affects roughly 10 to 20 in 100,000 children [1]. The underlying etiology of JIA is still unknown, and a lack of reliable biomarkers often delays diagnosis and makes the predicting of a child’s prognosis difficult. The IgM rheumatoid factor (RF) has been commonly used as a marker for the diagnosis of adult rheumatoid arthritis (RA) patients, but it has only been of value in diagnostic procedures for the small subset of JIA patients with polyarticular symptoms, often adolescents with small joint disease [1].

The anti-cyclic citrullinated peptide (anti-CCP) antibodies have been studied extensively in adult-onset RA and have proved to be highly specific for this disease (98%) [2]. Recent studies have revealed a possible role for anti-CCP antibodies in forecasting the severity of RA, with rapid radiographic progression [3-5]. Anti-CCP antibodies appear to be a predictive factor as they are accurate indicators that RA will occur within 3 years [6]. Meanwhile, for the diagnosis of JIA in childhood, the specificity of anti-CCP is extremely high (>95%) but its sensitivity is low, ranging from 2 to 14% [7-9]. Anti-CCP antibodies are present in the polyarticular, IgM RF-positive subset of JIA patients but are not valuable for the diagnosis of JIA in general [7-9]. Previous studies revealed that JIA patients who are positive for anti-CCP antibodies had severe clinical arthritis and radiological bone damage [9,10]. Anti-CCP may also be an independent predictor of radiological damage and disease progression [4,10]. Their simultaneous presence (anti-CCP and IgM-RF) may be an indication for more aggressive immunosuppressive treatments such as the use of biological therapy.

## Case presentation

**Figure 1:**
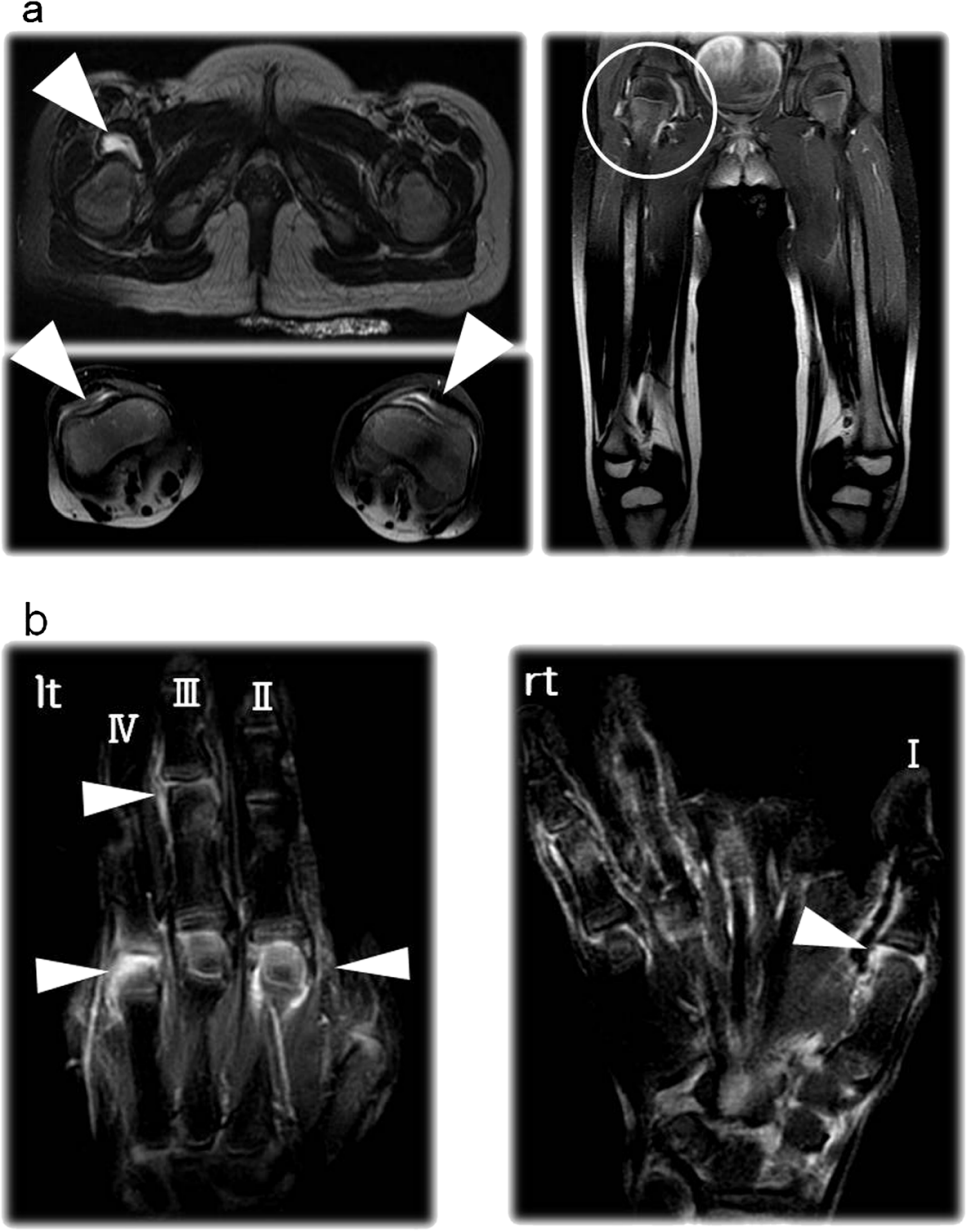
**a) MRI of the pelvis, lower extremities and hip and knee joints.** Fat SAT T2-weighted image demonstrates chronic inflammation with synovial thickening and/or fluid collection but without bone destruction. **b**) MRI of hands and fingers. Fat SAT T2-weighted image demonstrates chronic inflammation with synovial thickening and/or fluid collection but without bone destruction.

A Japanese girl, aged 3 years and 5 months, was referred to us for a 2-month history of bilateral knee, hand, and finger joint arthralgia with morning stiffness. She was born as a preterm infant at 28 weeks’ gestation by elective Caesarian section for fetal distress. Her birth weight was 872 g and she was under mechanical ventilation for 14 days. After that, her development was good and she remained healthy until 2 months before admission. Physical examination showed a well-appearing girl 93.2 cm tall and weighing 12.1 kg. Clinical examination revealed swelling and pain in bilateral knee joints, wrist joints, foot joints, hip joints and several metacarpophalangeal and proximal interphalangeal joints. She was unable to walk alone. Magnetic resonance imaging with T2 enhancement showed a synovial fluid collection without joint or bone destruction (Figure 1). The patient had moderate acute-phase responses, as indicated by the WBC count (15,000/μl), platelet count (67.3 × 104/μl), CRP (54 mg/l), and erythrocyte sedimentation rate (62 mm/hr). Other initial laboratory investigations showed increased levels of immunoglobulins G (22,860 mg/l), M (3,610 mg/l), and A (2,780 mg/l) as well as matrix metalloprotease-3 (MMP-3; 229.9 ng/ml). There were normal levels of ferritin (61.3 ng/ml), hyaluronic acid (36 ng/ml) and IgD (5 mg/l); liver function was also normal. Antinuclear antibodies (ANA) were positive (1:160). Anti-CCP antibody and IgM-RF were both positive (43.2 U/ml, 39.1 IU/ml respectively). The cut-off value for anti-CCP is 5.0 U/ml, and that for IgM-RF is 15.0 IU/ml. She had no iritis. She did not develop any of the other autoantibodies examined, and had no family history of rheumatic diseases or autoimmunity. Once the diagnosis of RF-positive polyarticular JIA was made, a non-steroidal anti-inflammatory drug (NSAID; ibuprofen, 30 mg/kg/day) was initially started. The Disease Activity Score (DAS) 28 is defined as 0.56 × √T28 + 0.28 × √S28 + 0.70 × ln(ESR) + 0.014 × general health (GH; patient assessment of disease activity using a 100-mm visual analogue scale with 0=best, 100=worst); our patient’s score was 6.39 [11].

**Figure 2:**
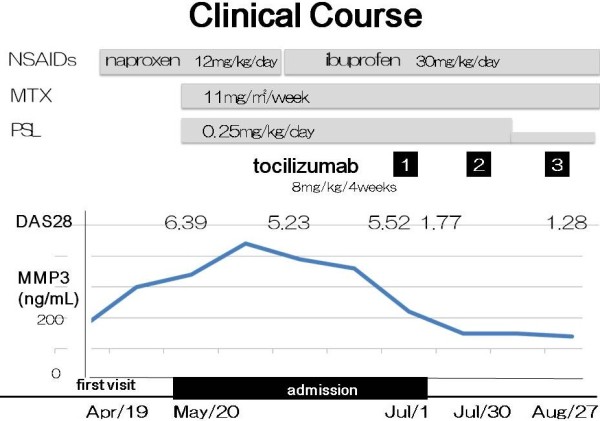
**Clinical course of the patient with anti-CCP antibodies and RF.** Tocilizumab was administered once every 4 weeks at a dose of 8 mg/kg. Disease activity score (DAS) 28 is defined as 0.56 × √T28 + 0.28 × √S28 + 0.70 × ln(ESR) + 0.014 x general health (GH; patient assessment of disease activity using a 100-mm visual analogue scale with 0=best, 100=worst).

Her symptoms were not controlled for the 3 weeks of the initial treatment. In succession, methotrexate (MTX, 10 mg/m2/week) and prednisolone (0.25 mg/kg/day) were added. During the next 6 weeks, acute-phase reactant levels did not decrease and DAS28 did not change (5.52) (Figure 2). Finally, tocilizumab was administered intravenously once every 4 weeks at a dose of 8 mg/kg. During this treatment period, the patient’s clinical symptoms began to improve rapidly. The level of acute-phase reactant returned to normal (CRP<0.1 mg/l), and the DAS 28 was 1.28 after the third administration of tocilizumab. After this dramatic improvement of the disease, we tapered and then stopped prednisolone treatment. The symptoms and signs of the disease did not recur for the next two years. The radiographic outcome was determined as normal and the serum concentration of MMP-3 was lower than 12.0 ng/ml. Her growth has been good. Anti-CCP antibody and IgM-RF titers were still positive; however, the values were lower (6.3 U/ml, 19.0 IU/ml respectively).

## Discussion

This is a report of the youngest child ever reported to develop both anti-CCP antibody positive and IgM-RF positive polyarticular JIA. Anti-CCP antibodies are now considered important markers for the diagnosis of RA in adults. In JIA, which is a very heterogenous groups of diseases compared to adult RA, the development of anti-CCP Abs is variable. High anti-CCP positivity is supposed to be related to highly RF-positive disease, and the RF-positive polyarticular type of JIA might be a pediatric equivalent of RA [9]. JIA mimicking adult RA has been reported in another 3-year old girl [12]. This child was reported to have severe, progressive, and erosive arthritis of the spine, shoulders, and extremities as well as pulmonary involvement. The patient had a high titer of RF, but an anti-CCP antibody was not measured. So, our patient is the youngest case with both anti-CCP antibody and IgM-RF to be reported worldwide.

She was born as a preterm infant. Interestingly, it is hypothesized that the pathogenesis of RA may be related to early life factors, such as birth weight and breastfeeding, which contribute to the development and shaping of the immune system [13]. Recent population-based case–control studies have explored a perinatal factor (e.g., breast feeding) in association with RA [14]. Yet our patient was fed cows’ milk.

Gestational age and prematurity may be related to infection susceptibility and to *in utero* maturation, but did not appear to be associated with RA incidence in either cohort [14-16]. Meanwhile, preterm birth was recently found to be a risk factor for systemic lupus erythematosus and related autoimmune conditions [17]. The immature immune system of a preterm neonate may be ill-suited to long-term alterations to the immune system. In our opinion, altered lymphocyte homeostasis in the thymus may lead to the development of autoimmunity.

It is suggested that JIA with both RF and anti-CCP positive antibodies is a more aggressive and severe disease that leads to joint damage and disability [10]. To prevent these poor outcomes, it appears prudent to formulate aggressive treatment strategies immediately. For our patient, the administration of tocilizumab was planned from an early stage. Evaluation of the efficacies of NSAID and MTX with steroid for a short duration of six weeks revealed no improvement. We then started the biologic and could conclude that the tocilizumab had brought about remission without the development of joint erosion and/or destruction at the 2-year follow-up. Serum MMP-3 is useful for assessment of inflammatory erosive arthritis [18]; here, the value of MMP-3 decreased with tocilizumab therapy in accordance with the remission of the disease. Although anti-CCP and IgM-RF were still positive, the values were lowered.

The importance of these antibodies in the evaluation of JIA and determination of possible treatment plans to prevent joint damage and disability was demonstrated. More studies are needed to resolve the precise roles of anti-CCP in JIA and to determine whether or not it works as a valuable marker for the selection of optimal treatments.

## Conclusions

This is a report of the youngest child known to develop anti-CCP antibody and RF double-positive polyarticular JIA who was successfully treated by tocilizumab. Early treatment of children with both RF and anti-CCP antibodies with a biologic may be optimal.

## Consent

Written informed consent was obtained from the patient and guardians for publication of this Case Report and any accompanying images.

## Abbreviations

JIA: Juvenile idiopathic arthritis; CCP: Cyclic citrullinated peptide; RA: Rheumatoid arthritis.

## Competing interests

None of the authors have any conflict of interest to report.

## Authors’ contributions

All authors worked in the ward of Hiroshima-City Hospital and conferred on the treatment and patient’s condition. The data was analyzed and interpreted mainly by KY and SS. All authors read and approved the final manuscript.
